# Surface effects on electronic transport of 2D chalcogenide thin films and nanostructures

**DOI:** 10.1186/s40580-014-0018-2

**Published:** 2014-05-31

**Authors:** Yeonwoong Jung, Jie Shen, Judy J Cha

**Affiliations:** 1 Department of Mechanical Engineering and Materials Science, Yale University, New Haven, CT USA; 2 Energy Science Institute, Yale University West Campus, West Haven, CT USA

**Keywords:** Two-dimensional materials, Transition metal chalcogenides, Topological insulators, Bi_2_Se_3_, MoS_2_, Surface effects

## Abstract

The renewed interest in two-dimensional materials, particularly transition metal dichalcogenides, has been explosive, evident in a number of review and perspective articles on the topic. Our ability to synthesize and study these 2D materials down to a single layer and to stack them to form van der Waals heterostructures opens up a wide range of possibilities from fundamental studies of nanoscale effects to future electronic and optoelectronic applications. Bottom-up and top-down synthesis and basic electronic properties of 2D chalcogenide materials have been covered in great detail elsewhere. Here, we bring attention to more subtle effects: how the environmental, surface, and crystal defects modify the electronic band structure and transport properties of 2D chalcogenide nanomaterials. Surface effects such as surface oxidation and substrate influence may dominate the overall transport properties, particularly in single layer chalcogenide devices. Thus, understanding such effects is critical for successful applications based on these materials. In this review, we discuss two classes of chalcogenides – Bi-based and Mo-based chalcogenides. The first are topological insulators with unique surface electronic properties and the second are promising for flexible optoelectronic applications as well as hydrogen evolution catalytic reactions.

## 1 Introduction: renewed interest in 2D chalcogenides

**Figure 1:**
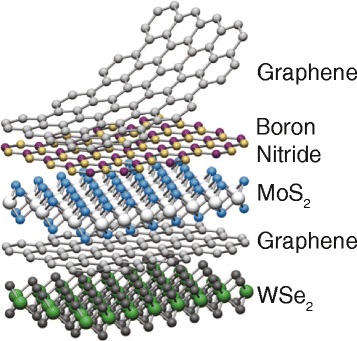
**Van der Waals heterostructures, stacking 2D chalcogenide single layers, graphene, and hBN together to form a crystal.** These heterostructures present opportunities to tailor electronic properties, not found in their bulk counterparts. Reprinted with permission from Ref 17.

Following the success of graphene [1–4], layered materials that can be peeled down to a single layer have received tremendous attention for their novel electronic, chemical, and mechanical properties. These include atom-thick, hexagonally arranged two-dimensional (2D) sheets such as hexagonal boron nitride (hBN) [5–7], silicene [8,9], and germanene [10,11], and layered oxides and chalcogenides [12] such as MoS_2_ [13], WSe_2_ [14], Bi_2_Se_3_ [15], and Bi_2_Te_3_ [16] to name a few. In chalcogenides, a few atomic layers are covalently bonded to make a molecular layer, and the molecular layers stack together to form crystals via the relatively weak van der Waals interaction. Van der Waals heterostructures (Figure 1) using single layer chalcogenides, graphene, and hBN as basic building blocks present exciting opportunities to tailor electronic band structures for specific applications [17].

The electronic properties of single layer chalcogenides deviate from the bulk. For example, the topological surface state will be gapped if Bi_2_Se_3_ is less than 5 layers thick [18], and a single layer MoS_2_ becomes a direct bandgap semiconductor instead of an indirect bandgap semiconductor [19–21]. As these chalcogenides are synthesized to single- or a few-layer thick films or nanosheets, surface effects (both desired and undesired) will be enhanced. In perfect crystals, the chalcogenide nanostructure is terminated with basal planes that are inert with saturated bonds. However, in reality, crystal defects and surface absorbates cannot be avoided, leading to surface trap sites or changes in mobility and carrier density of the sample. Thus, the unforeseen surface/parasitic effects on electron transport on 2D chalcogenide thin films and nanostructures should be studied carefully. This review will look at how surface or parasitic effects play a role in 2D chalcogenide nanomaterials. Two chalcogenide systems, Bi-based selenides (topological insulators) and Mo-based sulfides (semiconductors and hydrogen evolution catalysts), will be reviewed in detail. Detailed reviews on synthesis and basic electronic properties of 2D chalcogenides can be found elsewhere [22–24].

## 2 Graphene

Before delving into the chalcogenide systems, we briefly cover the surface effects on graphene transport. We focus our attention on substrate-induced charge fluctuations and surface functionalization.

### 2.1 Substrate effects: SiO_x_ substrate, suspended graphene, and hexagonal boron nitride

**Figure 2:**
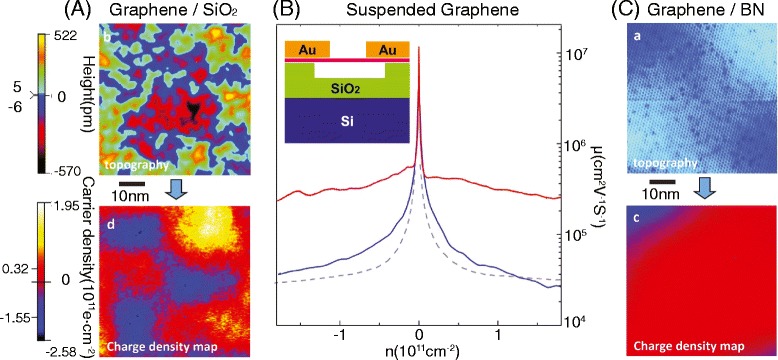
**Substrate effects on graphene transport. (A)** STM topographic (top) and charge density (bottom) maps of graphene on SiO_2_. The surface is rough due to the substrate. Inhomogeneous charge density fluctuations are observed. **(B)** Mobility of graphene as a function of carrier density for graphene on SiO_2_ (dotted gray), suspended graphene before (blue) and after (red) current annealing. Current annealing of suspended graphene increases mobility as surface adsorbates desorb from graphene. **(C)** STM topographic (top) and charge density (bottom) maps of graphene on hBN. Unlike the STM results of graphene on SiO_2_, the topography shows a flat surface with Moiré patterns. Charge fluctuations are greatly reduced in the graphene/hBN case. **(A, C)** Reprinted with permission from Ref. 26. (B) Reprinted with permission from Ref. 29.

Electron transport on graphene is subject to microscopic perturbations that can cause backscattering, greatly decreasing the electron mean free path as well as mobility. In the case of high quality graphene sheets without defects, impurities are induced by extrinsic sources. Scanning tunneling microscopy (STM) results show inhomogeneous electron density fluctuations caused by charge-donating impurities at the interface between graphene and SiO_2_ substrate (Figure 2A) [25,26]. These substrate-induced impurities can be high enough to create standing waves in graphene due to enhanced backscattering [27]. One way to eliminate the substrate effects in graphene transport is to remove the substrate entirely. Several groups reported free-standing graphene sheets suspended over a trench [28,29]. Suspended graphene exhibits corrugation in the out-of-plane dimension, thus becoming thermodynamically stable [28]. Electron transport on the suspended graphene device (Figure 2B) shows ultrahigh electron mobility, greater than 200,000 cm^2^/Vs at the electron density of ~2 × 10^11^ cm^−2^ [29]. This is a dramatic improvement compared to mobility values reported in graphene devices fabricated on conventional substrates [30]. Current annealing can further improve the mobility in suspended graphene devices as impurities on graphene can desorb during annealing [29]. Nearly ballistic transport can be observed in suspended devices [31]. The dramatic improvement in transport for suspended graphene devices compared to graphene/SiO_2_ devices highlights how substrate or parasitic effects can completely dominate transport in these thin nanodevices.

Although suspended graphene has distinct advantages over graphene on conventional substrates in terms of transport properties, it is fragile and difficult to handle. Thus, finding an alternative substrate to SiO_2_/Si while keeping the same transport properties as suspended graphene devices is essential for any successful device applications. Hexagonal boron nitride (hBN) is an appealing substrate because its surface is atomically smooth, its lattice mistmach to graphene is small (~1.7%), and it has a large bandgap (5.97 eV) [6,32]. According to STM studies [26], graphene on hBN is very flat so that microscopic Moiré patterns arising from the relative mis-orientation between the graphene and hBN lattices can be observed. Gate-dependent *dI/dV* spectra of graphene on hBN from STM studies exhibit a significant reduction in local microscopic charge inhomogeneity (Figure 2C). The mobility of graphene on hBN improves by ~ 3 times compared to SiO_2_-supported graphene samples [32].

### 2.2 Surface functionalization

The surface of graphene can be functionalized with chemical species for effective Fermi level control and bandgap opening [33]. Surface functionalization includes partial plasma etching [34,35], surface absorbates [36], and covalent bonding with small molecules [37,38]. Although effective in modifying the band structure of graphene such as p- or n-type doping and bandgap opening [39–41], often these functional groups lead to increased scattering for electron transport, dramatically decreasing the mobility and the mean free path length. For room temperature applications such as graphene as transparent electrodes or conducting electrodes for Li-ion batteries [30,42–44], the degradation in mobility may not be an issue as long as the conductivity is high. However, for low-temperature transport for quantum or nanoscale effects, surface functionalized graphene may not be suitable.

## 3 Bi_2_Se_3_, Bi_2_Te_3_: topological insulators

Traditionally well-known thermoelectric materials, such as Bi_2_Te_3_ and Bi_2_Se_3_, have been recently classified as a new quantum matter called topological insulators [45–47]. The most unique aspect of topological insulators is the robust, spin-polarized conducting surface state that encapsulates topological insulators. The topological surface state serves as a platform to study fundamental quasi-particle phenomena such as Majorana fermions [48,49] and magnetic monopole-like behavior [50] as well as to explore future electronics such as quantum computing and spintronics [51,52]. To maximize the topological surface state for transport, often Bi_2_Te_3_ and Bi_2_Se_3_ are made into nanostructures or thin films [24]. Unfortunately, experimental studies report degradation of the topological surface state transport property due to environmental effects. Current challenges for Bi_2_Se_3_ and Bi_2_Te_3_ are minimizing these detrimental environmental effects as well as improving the intrinsic materials properties so that the topological surface states can be more easily revealed and manipulated. Here, we review three such effects.

### 3.1 Strain from substrates

Bi_2_Se_3_ and Bi_2_Te_3_ thin films have been successfully grown on a number of substrates such as Si, sapphire, graphene, and on mica by both molecular beam epitaxy and chemical vapor deposition [53–57]. Their topological surface states were clearly observed by angle-resolved photoemission spectroscopy [58,59]. STM studies also showed the presence of the surface states in these thin films by confirming the Dirac dispersion [60]. However, the quantum nature of the surface state was not easily revealed by transport measurements in thin films. This is mainly because the mobility of the surface state was low, as evidenced by the clear weak anti-localization effect and the lack of Shubnikov-de Hass (SdH) oscillations in transport [61,62]. Weak anti-localization occurs in diffusive regime where the electrons go through many scattering events. Thus, it indicates short mean free paths, usually around 300 – 500 nm for Bi_2_Se_3_ and Bi_2_Te_3_ [63,64]. Only a few studies show thin films exhibiting 2D SdH oscillations, which are attributed to high mobility surface electrons [65]. The reason for the low mobility of topological surface electrons in thin films is likely due to the strain from the substrate. Because Bi_2_Se_3_ and Bi_2_Te_3_ are layered materials with weak van der Waals interactions, they can be grown on substrates with a relatively large lattice mismatch. The strain induced from the lattice mismatch as well as charge fluctuations from the substrate may be the reason for the low mobility of the topological surface states.

### 3.2 Surface oxidation

Residual bulk carriers due to crystal defects such as Se vacancies in Bi_2_Se_3_ and anti-site defects in Bi_2_Te_3_ have hindered transport studies of topological surface states greatly. Compensation charge doping using Sb or Cu is effective in suppressing the bulk carriers [66–69]. However, it was soon realized that different size samples from the same growth batch showed different carrier densities; smaller samples showed higher carrier densities [70]. This finding hints to additional surface effects playing a role in transport. In particular, Bi_2_Se_3_ nanoribbon studies showed that the surface of Bi_2_Se_3_ was oxidized quickly by observing BiO_x_ and SeO_x_ peaks using X-ray photoemission spectroscopy [71]. The same nanoribbons showed two different carrier densities. The Hall carrier density was much higher than the density obtained from SdH oscillations. Because the Hall measurements include all carriers while the SdH measurements sample only the high mobility carriers, the discrepancy in carrier densities was attributed to additional, low-mobility carriers induced by the environmental effects including the surface oxidation. The surface oxidation induces additional scattering events for the topological surface state where the oxidized radicals can act as trap sites or induce charge fluctuations.

### 3.3 Fermi level pinning

**Figure 3:**
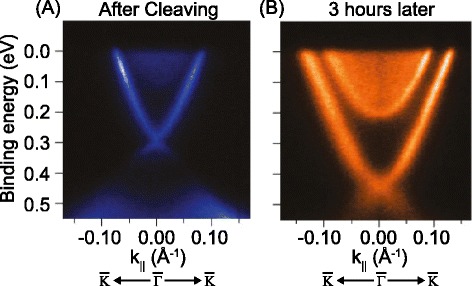
**Band structure measurements of Bi**_**2**_ **Se**_**3**_ **from angle-resolved photoemission spectroscopy. (A)** shows the band structure of freshly cleaved Bi_2_Se_3_ surface. The surface state is clearly visible. **(B)** Band structure of the same surface, after left in vacuum for three hours. The Dirac point shifted down. In addition, a sharp rim is observed around the conduction band edge after three hours. This indicates emergence of a trivial 2D electron gas. Reprinted with permission from Ref. 72.

Time-dependent angle-resolved photoemission spectroscopic studies show the emergence of a topologically trivial 2D electron gas forming at the surface of Bi_2_Se_3_ as a function of time (Figure 3) [72]. The 2D electron gas then coexists with the topological surface state, contributing to transport. This complicates the interpretation of transport studies because simply verifying the 2D nature of the carrier in transport is not sufficient to distinguish the topological insulator surface states from the trivial 2D electron gas. Indeed, a theoretical model for weak anti-localization effects in thin Bi_2_Se_3_ or Bi_2_Te_3_ films shows that contribution from the 2D electron gas can lead to uncertainty related to the value of α [73], the parameter whose value can indicate how many topologically non-trivial states exist in the thin film [74]. Earlier studies of SdH oscillations that were 2D in nature may have to be studied more carefully to distinguish the effect of the 2D electron gas [75,76]. The formation of the 2D electron gas is attributed to the Fermi level pinning. In addition, the fact that, overtime, multiple 2D electron gases emerge also hints to environmental effects playing a role.

### 3.4 How to reduce parasitic effects

The most logical way to reduce parasitic effects for topological insulator Bi_2_Se_3_ and Bi_2_Te_3_ has been to coat the thin films or nanostructures with a protective layer that does not interfere with the topological surface state [69]. For Bi_2_Se_3_, *in situ* Se coating after the growth seems particularly effective in keeping the carrier density low and mobility high. The Se layer itself is insulating, thus does not affect transport significantly. Careful Aharonov-Bohm oscillations were carried out after *in situ* Se coating on Bi_2_Se_3_ narrow nanowires [68]. The coating method, however, may not be feasible for proximity effect studies where topological insulators need to be interfaced directly with either superconductors or ferromagnetic insulators [77,78]. In such cases, the superconducting or ferromagnetic layers can be grown on top of topological insulators to serve as a protective layer. Care must be taken to ensure that the subsequent growth do not damage the topological insulators.

## 4 MoS_2_ : indirect- to direct- gap semiconductor

**Figure 4:**
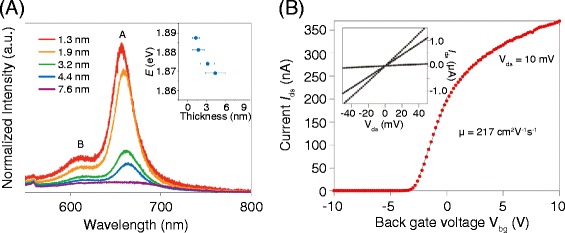
**Optical and electrical properties of mono-to-a few layer MoS**_**2**_ **. (A)** Photoluminescence spectra of MoS_2_ thin films with varying layer thicknesses. Inset shows the energy of the exciton peak, A, as a function of layer thickness. **(B)** Room-temperature transport characteristics of a monolayer MoS_2_ FET. Inset shows drain-source current vs. drain-source bias under varying back gate voltages. **(A)** Reprinted with permission from Ref 81. **(B)** Reprinted with permission from Ref 13.

Unlike graphene, semiconducting characteristics of 2D chalcogenides, manifested by a bandgap and high on/off current ratio, make them attractive for electronic devices such as field-effect-transistors (FETs). In addition, the layer thickness-dependent tunable bandgap and its indirect-to-direct transition inherent to these materials suggest a novel opportunities for optical applications [19,79–81] (Figure 4A, enhanced photoluminescence in single layer MoS_2_). For successful applications, charge carrier transport of the 2D chalcogenides needs to be studied carefully, which is determined by intrinsic materials properties as well as extrinsic materials preparation methods. Large uncertainty in both the intrinsic and extrinsic factors often leads to a huge variation in the performance of electronic devices based on these materials. For example, FETs based on mechanically exfoliated mono- to a few-layer thick MoS_2_ generally present high carrier mobility, some approaching the theoretical limit set by phonon scattering [13,20,82–85] (Figure 4B). Meanwhile, chemically synthesized MoS_2_ FETs are often limited with poor carrier mobilities, over an order of magnitude lower than the theoretical value [86–88]. Amongst a number of such variables, the surface property of the 2D chalcogenides is believed to play a critical role in their carrier transport due to the large surface areas, as in the case of graphene electronics we covered earlier. Indeed, the conductivity of few-layer thick MoS_2_ is found to be highly sensitive to ambient environments [89,90], indicating the important role of its surface states for carrier transport.

In this section, we review the surface and interfacial effects on transport properties of 2D chalcogenides. We focus our discussion on MoS_2_, one of the most explored 2D chalcogenides, and discuss the followings: (1) extrinsic interfacial effects on MoS_2_ in contact with other medium such as metal contacts and dielectric substrates, and (2) intrinsic surface properties of MoS_2_ such as surface, electronic, and crystal defects.

### 4.1 Extrinsic interface effects: MoS_2_-metal and MoS_2_-dielectrice interface

In this section, we exclusively deal with mechanically exfoliated mono-to a few-layer thick MoS_2_ nanoflakes. Choice of contact metals for MoS_2_ plays an important role in determining the carrier type (p- or, n-) and the performance of MoS_2_ FETs. Das et al., have systematically studied various metal contacts for monolayer MoS_2_ FETs, and found metals with low work functions form lower contact resistance than high work function metals [91]. As a result, very high FET mobility of ~ 700 cm^2^/Vs is realized when Scandium (Sc) was used as a contact metal. The n-type characteristics were unaffected by the choice of metal contacts in this study. In contrast, Fontan et al., showed that MoS_2_ FETs can display both n- or p-type behaviors depending on the choice of contact metals [92]. Gold (Au) contacts showed n-type characteristics while Palladium (Pd) contacts showed p-type characteristics. This was attributed to the local Fermi energy pinning of MoS_2_ at the MoS_2_/Pd interface due to the lowered Fermi energy level of Pd caused by the chemical interaction between Pd and MoS_2_. A computational study found that Au forms a tunnel barrier contact while Titanium (Ti) forms a more electron injection-efficient low resistance Ohmic contact on a monolayer thick MoS_2_ [93]. These studies point to the importance of characterizing the MoS_2_-metal interface carefully, where transport characteristics may be dominated more by the interface effects, rather than the intrinsic MoS_2_ properties.

In addition to the MoS_2_-metal contact issues, dielectric substrate effects on MoS_2_ are also important. A first-principle calculation has revealed that the electronic properties of a monolayer MoS_2_ are greatly affected by underlying SiO_2_ substrate. Presence of oxygen dangling bonds at the SiO_2_/MoS_2_ interface can cause the downward shift of Fermi energy below the valence band maximum, thus converting the originally n-type MoS_2_ to p-type MoS_2_ [94]. Using high k-materials such as aluminum oxide (Al_2_O_3_) or hafnium oxide (HfO_2_) as gate dielectric layers instead of SiO_2_ can improve the performance of MoS_2_ FET without converting carrier types [91,95].

### 4.2 Intrinsic surface effects: structural and electronic defects in CVD- synthesized MoS_2_

**Figure 5:**
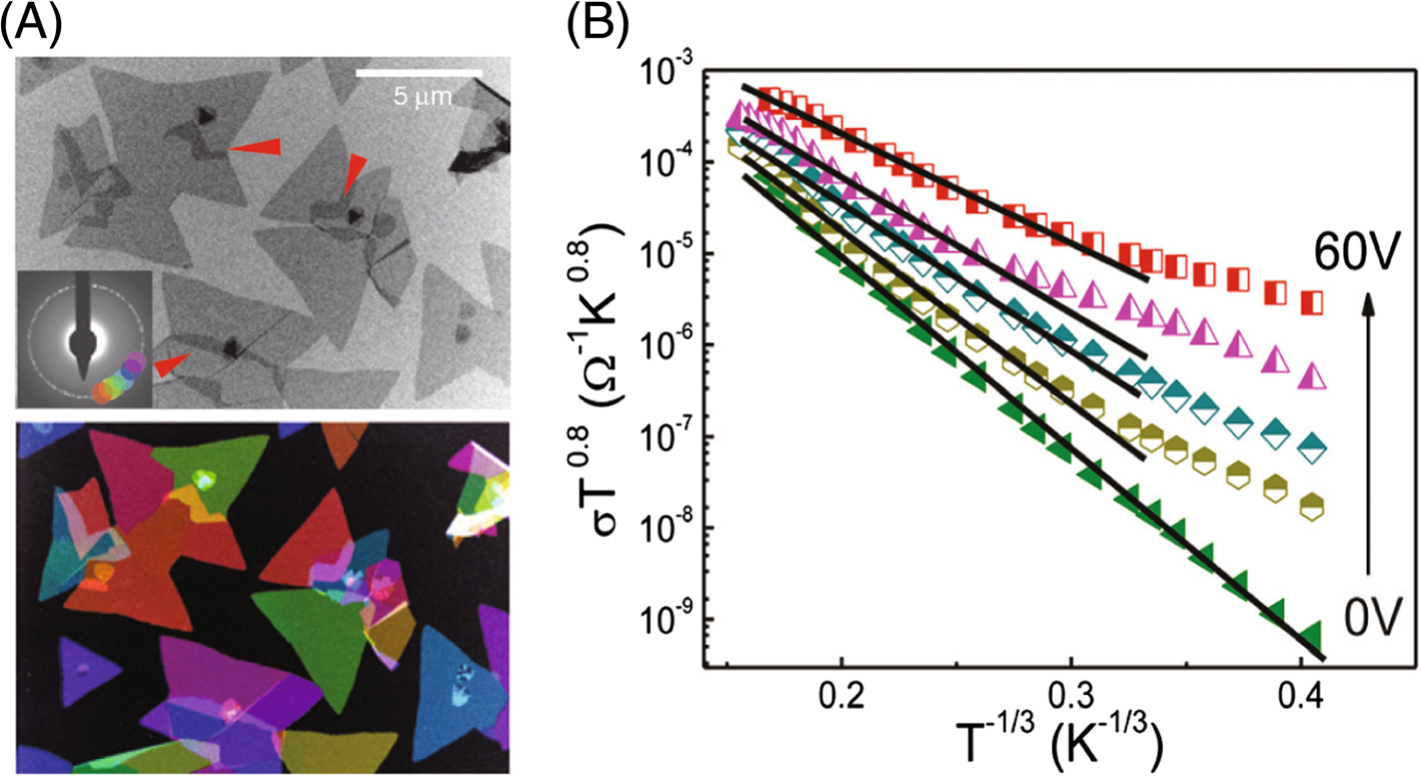
**Intrinsic structural defects and defect dominated transport in MoS**_**2**_ **. (A)** Bright-field transmission electron microscope (TEM) image (top) and its corresponding colored dark-field image (bottom) show irregularly shaped polycrystalline grains of MoS_2_. **(B)** Temperature-dependent conductivity of a few-layer MoS_2_ FET under various back gate voltages. The linear fits indicate 2-D variable-hopping transport in MoS_2_. **(A)** Reprinted with permission from Ref 100. **(B)** Reprinted with permission from Ref 97.

Zhu et al., have systematically studied the carrier transport properties of MoS_2_, synthesized by chemical vapor deposition (CVD) [96]. They found that CVD-grown MoS_2_ possesses a significant amount of band tail trapping states, which severely impair FET performances. Because of the trapped charges, measured mobility values largely fall short of the true band mobility predicted by theory. This finding agrees well with earlier studies of the low-temperature FET characterizations of MoS_2_, which revealed highly localized electronic states and trap-assisted 2D variable hopping-range transport in MoS_2_ [97,98]. The presence of intrinsic structural defects, such as point defects, dislocations, and grain boundaries, in CVD-grown monolayer MoS_2_ have been confirmed by atomic resolution imaging techniques and they were correlated with localized mid-band gap states [99,100] (Figure 5A). Strong anisotropy in electron transport directions due to defects such as grain boundaries is observed [100]. In addition, sulfur vacancies in MoS_2_ are found to introduce localized donor states inside the bandgap, thus are responsible for the n-type behavior of MoS_2_ as well as the hopping-dominated transports [101] (Figure 5B). A theoretical study also suggests that the presence of intrinsic atomic vacancies in MoS_2_ leads to localized mid-gap states that act as scattering centers, thus significantly reducing conductance [102]. The decrease of conductance depends on the type and the concentration of the defects.

Besides their effect on carrier transport efficiency, the intrinsic structural defects of MoS_2_ can also affect the MoS_2_-metal contact properties as well as charge carrier types. McDonnell et al., have revealed that the intrinsic defects of MoS_2_ dominate the MoS_2_/metal contact resistance, resulting in a low Schottky barrier independent of contact metal work functions [103]. In addition, they showed that MoS_2_ can exhibit both p-type and n-type at different positions on a same sample, which is attributed to variations in the local stoichiometry of MoS_2_ due to surface defects. Electrical and materials characterizations revealed that the regions of n-type possess ~1:1.8 stoichiometry in Mo:S and local S vacancies are responsible for the n-type. Meanwhile, the p-type regions possess a stoichiometry of ~1:2.3, indicating they are either S-rich (intercalates, or interstitials) or Mo-deficient. The study further emphasizes the critical role of the surface states of MoS_2_ on carrier transport properties and strengthens the importance of eliminating un-wanted surface properties for improved carrier transports.

### 4.3 Ways to eliminate interface and surface effects

Since the surface/interface properties of MoS_2_ contribute tremendously to the carrier transport in MoS_2_-based electronic devices as reviewed above, the effective elimination of unwanted surface/interface effects is critical for improving transport properties. Efforts have been focused on improving the interfacial properties - MoS_2_-metal contact and MoS_2_-substrate interfaces. External dopants were introduced to MoS_2_ FETs to lower the Schottky barrier and contact resistance at the MoS_2_-metal interface. Degenerate n-doping of MoS_2_ has been realized by using Potassium, a material of high electron affinity, as a dopant [104]. Molecular doping approaches, such as using polyethyleneimine (PEI) molecules as n-dopants, have also been proven to improve FET characteristics [105,106]: ~50-70 % improvement in ON current and FET mobility. Substantial efforts have also been made in improving the MoS_2_-dielectric substrate interface properties. Much of these have been focused on using better dielectric materials [83,95,107]. Bao et al., have demonstrated the highest room-temperature FET mobility of ~ 470 cm^2^/Vs on polymethyl methacrylate (PMMA)-supported MoS_2_ FETs, > 10 times higher than the mobility of comparable devices on SiO_2_ substrates [83]. This superiority is attributed to the effective dielectric screening from the PMMA [83,108].

In comparison to the efforts to eliminate extrinsic effects on MoS_2_ transport properties, not much progress has been made in improving the intrinsic properties of MoS_2_ for better carrier transport. A limited number of works based on post-growth approaches have been reported. For example, surface-passivated MoS_2_ FETs with passivation layers (eg. PMMA [89] or Silicon nitride (SiNx) [109]) have demonstrated better transport characteristics, attributed to the effective protection of MoS_2_ against the adsorption of environmental molecules rather than the alteration of intrinsic surface/structural properties of MoS_2_. More promising and fundamental approaches lie in developing effective growth schemes that could produce MoS_2_ with intrinsically high surface qualities. This remains largely unexplored at present and more systematic work is needed in this direction.

## 5 Conclusion

The progress and renewed interest in 2D chalcogenides have been remarkable. With continued improvement in 2D chalcogenide synthesis and a myriad of possible van der Waals heterostructures with 2D chalcogenides as basic building blocks, future of 2D chalcogenides for fundamental studies as well as optoelectronic applications is promising. As we focus on mono- to a few-layer thick chalcogenide thin films and nanosheets to enhance desirable nanoscale or surface effects, it becomes increasingly important to distinguish true intrinsic materials properties from extrinsic environmental effects such as contact metal issues, surface defects such as oxidation or vacancies, and interface-induced impurities or trap sites. Systematic transport and materials characterizations are critical to reveal these parasitic effects. This review highlighted several experimental and theoretical works on extrinsic surface and interface effects on electron transport properties of Bi-based chalcogenide topological insulators and Mo-based sulfide semiconductors. Research efforts on these secondary environmental effects must be concurrent with fundamental intrinsic property studies to minimize any possible misinterpretation of data and to ensure rapid progress of the field.
